# Predictors of physical activity among pregnant women in Harare, Zimbabwe

**DOI:** 10.1371/journal.pgph.0003470

**Published:** 2025-01-06

**Authors:** Anotida R. Hove, Eleanor N. Sithambuli, Wethembekile P. Ncube, Shalom R. Doyce, Sandra N. Mukwekwe, Tariro Mapuranga, Kudakwashe Tirivanhu, Dixon Chibanda, Jermaine M. Dambi

**Affiliations:** 1 Department of Rehabilitation Sciences, Faculty of Medicine and Health Sciences, University of Zimbabwe, Avondale, Harare, Zimbabwe; 2 Mental Health Unit, Faculty of Medicine and Health Sciences, University of Zimbabwe, Avondale, Harare, Zimbabwe; Babcock University, NIGERIA

## Abstract

The extensive benefits of physical activity (PA) are well known. However, PA participation among pregnant women remains low. This study evaluated PA levels and associated factors, including barriers and facilitators in pregnant women in Harare, Zimbabwe. This cross-sectional study recruited 517 pregnant women receiving antenatal care across ten primary healthcare clinics. Data were collected using the Pregnancy Physical Activity Questionnaire (PPAQ), Exercise Benefits and Barriers Scale (EBBS) and EQ-5D-5L. We used descriptive statistics and binary logistic regression for analyses. The mean age of the study participants was 26.1 (±5.9) years. 89% of the pregnant women did not meet the WHO recommendations of 150 minutes of moderate-intensity PA. For women who were active, most engaged in moderate intensity (41.9%) PA and household activity (51.1%). The most perceived barriers and facilitators to PA were reported as exercise environment and life enhancement, respectively. Lower education (AOR 5.24 [1.69: 16.19]), multigravida (AOR .47 [.31: .42]), not exercising pre-pregnancy (AOR 2.02 [1.30: 3.13]), perceived decreased current PA level (AOR 2.04 [1.22: 3.43]) and not being advised by a doctor on exercise (AOR 2.05 [1.04: 4.04]) were associated with physical inactivity. Physical inactivity is endemic among Zimbabwean pregnant women, there is a need for bespoke and contextualized interventions. Implementing supervised and group-based antenatal exercise classes must be considered.

## Introduction

Physical inactivity is a significant public health concern, with [1, 2]. The burden of physical inactivity extends beyond its negative consequences on an individual’s health to the global economy. For example, the global economic burden of physical inactivity is estimated at US$ 27 billion per annum [3]. To realize physical activity-associated health benefits, the World Health Organization recommends at least 150 minutes of moderate-intensity weekly exercise for adults, including pregnant women [4]. Yet, over 27% of adults globally and up to 91.6% of pregnant women from Africa do not meet the WHO recommendations for physical activity (PA) [4, 5]. Pregnant women are often less active than their non-pregnant counterparts and tend to decrease their activity levels during pregnancy [1, 6]. For instance, a South African cohort study [N = 332] showed PA decreases with pregnancy progression, particularly from the second to the third trimester, attributable to the physical and physiological changes during pregnancy [6, 7]. Physiological changes that occur during pregnancy affect the cardiac (increased heart rate), renal, respiratory (increase in oxygen demand, breathlessness), endocrine and skeletal (lower bone density) systems [8]. These physiological changes alter pregnant women’s tolerance to exercise, hence the decline in PA. Also, other pregnancy-related physical and psychosocial changes predispose pregnant women to a high risk of physical inactivity [1, 2, 9]. For example, pregnancy-specific psychological factors such as fear of miscarriage and physical factors such as frequent bodily pain and aches, nausea, tiredness and increased stomach size may contribute to a reduction in physical activity levels [1, 2, 9, 10].

Contrary to the common belief that women in low-income countries have a high level of PA, considering their high physical workload even during pregnancy, PA during pregnancy is low [6]. The most commonly cited reasons among African pregnant women for being physically inactive include a lack of knowledge/information, time pressure and low social support [5]. In Sub-Saharan Africa, sociodemographic factors such as low maternal education, resource limitations, poverty, religion, and multi-parity are associated with low PA during pregnancy [11–14]. Also, misconceptions that exercising during pregnancy is linked with miscarriage are highly prevalent in women with little or no formal education [15]. Knowledge-related barriers are salient predictors of physical inactivity in pregnant women in low-income settings [11, 16]. Knowledge, or the lack thereof, influences pregnant women’s capability and motivation to engage in PA [17]. In a qualitative study done on South African pregnant women, a lack of knowledge regarding the type and intensity of PA evolved as a major barrier to regular physical activity engagement [18]. Unfortunately, low education is interlinked with poverty, which is inherently linked to a lack of access to equitable healthcare (e.g. advice on safe PA engagement practices) and PA resources (e.g. access to the gym or safe exercise spaces) [1, 5].

Physical activity is a modifiable risk factor contributing to maternal and fetal health [9]. Pregnancy is an ideal time to adopt and maintain healthy lifestyle habits due to the mother’s interest in the child’s health [19]. Regular PA involved in pregnancy comes with psychological benefits, which include improved mood, self-esteem, reduced depressive symptoms, better health-related quality of life and prenatal anxiety [20–22]. The low PA engagement occurs at a time when one benefits the most from being physically active. Pregnant women who are not sufficiently engaging in PA are at risk of poor maternal health (e.g. high risk of gestational obesity and diabetes, high blood pressure, urinary incontinence, preeclampsia), increased risk of birth complications (e.g. prolonged labor, emergency caesarean section), and adverse neonatal outcomes (e.g. preterm delivery and low birth weight) [1, 9, 10, 12, 20, 23–25].

Although multiple studies have explored the prevalence of PA in pregnant women, few of these studies have quantitatively explored the barriers and facilitators associated with PA engagement. The evidence gap is even more glaring in pregnant women in Sub-Saharan Africa. For example, in a systematic review exploring PA levels in pregnant women, only three out of the 44 studies included were from Africa [26]. Consequently, there is a need for studies that holistically explore physical activity and its determinants, including identifying barriers and facilitators. Understanding contextualized barriers and facilitators to PA participation in pregnancy is essential to inform the development and implementation of bespoke interventions to increase PA levels in pregnant women [16]. Therefore, given the need to contextualize PA engagement behaviors and practices, the objective of this study was to determine the PA level, patterns, perceived barriers, and facilitators to PA engagement in pregnant women to improve maternal and pregnancy outcomes among Zimbabwean pregnant women.

## Materials and methods

### Ethics statement

Ethical approval to conduct the study was obtained from the Joint Research and Ethics Committee for the University of Zimbabwe Faculty of Medicine and Health Sciences & Parirenyatwa Group of Hospitals (JREC/149/2023). Institutional approval was granted by the City Council of Harare to access polyclinics where the study was conducted. Participants were approached in the waiting areas, informed about the study and its objectives, and offered to participate. They were treated as autonomous agents and only recruited into the study upon signing the consent form voluntarily. Confidentiality was maintained as each participant was assigned a participant identification number.

### Study design and setting

This cross-sectional study was conducted in the capital city of Zimbabwe, Harare. Participants were recruited from 10 randomly selected urban polyclinics; the City of Harare runs 12 polyclinics. The Zimbabwean healthcare system is divided into primary, secondary, and tertiary healthcare tiers. Primary clinics and polyclinics constitute the primary healthcare tier and provide entry-level healthcare. The polyclinics are located within residential suburbs to ensure easy access to healthcare for the urban population. Polyclinics offer primary health care, family health, and outpatient medical services, including antenatal care, immunizations, and maternity care services. Most of the polyclinics are in high-density areas where large numbers of pregnant women are attended to daily. Polyclinics in high-density areas receive approximately 30–50 visits daily from pregnant women attending subsequent visits.

### Participants

We recruited voluntarily consenting pregnant women over the age of 18 years who were able to understand either English, Shona (a local Zimbabwean language), or both. Participants were excluded from the study if they were acutely ill and requiring emergency care, were in active labor, intoxicated, had been advised by a doctor or health professional not to engage in PA due to contraindications (e.g. gestational hypertension, vaginal bleeding), or had any condition that could affect their ability to give consent and participate in the study.

### Sample size calculation

The sample size was calculated using estimates from an almost similar study in Ethiopia, yielding a 20.3% (p_0_) prevalence in physical activity [12]. Assuming Zimbabwean pregnant women are more physically active, they are more likely to be involved in manual tasks (p_1_ = 0.25). At least 497 pregnant women were required at α = .05 and β = .80, assuming 25% extreme scores. The sample size was estimated using STATISTICA (Version 13).

### Data collection procedure

Participants were approached as they waited to receive antenatal services from the nursing staff and conveniently recruited to participate in the study. They were informed about the study and its objectives and then offered a chance to participate. Once a participant signed the consent form, the demographic and physical activity questionnaires were interviewer-administered to ensure more reliable and valid responses. Afterwards, participants either self-completed the remaining questions or the interviewer proceeded with the interview. Where respondents chose self-completion, the researchers were always in proximity to clarify if the participants did not understand any questions. All data was collected electronically using KoboCollect software programmed on Samsung computer tablets.

### Outcome measures

**The Pregnancy Physical Activity Questionnaire (PPAQ)** was used to measure the level of physical activity in pregnant women. This questionnaire comprises 33 items requiring respondents to approximate the time spent on household, occupational, transportation, and sport/recreational activities. Items are weighted by duration and intensity; the weekly energy expenditure/metabolic equivalent of task (METs-h-week) per item/domain is calculated by multiplying duration and intensity. The total activity is the summation of the product of duration and intensity. Using the total METs criterion, activity levels are classified as sedentary, light, or vigorous. The PPAQ is an extensively used pregnancy-related PA outcome measure with evidence of robust psychometric performance [27]. Also, the PPAQ has excellent cross-cultural validity performance and has been used in settings comparable to the current study, i.e., it has been used in Nigeria, Ethiopia, and South Africa [12, 28, 29]. This questionnaire was formally translated by professionals into Shona using forward-backwards translation and culturally adapted to suit the Zimbabwean setting. A pilot study was conducted, which confirmed the tool’s conceptual- and semantic equivalency to the target language.

**The Exercise Benefit Barriers Scale (EBBS)** is a 26-item questionnaire measuring participants’ perceived barriers and benefits of exercise [30]. It consists of 19 benefits statements and seven (7) barrier statements. The respondent must respond to each statement based on a 4-point Likert scale with these responses: strongly disagree, disagree, agree, strongly agree. The responses for benefits are weighted from 1 to 4, respectively, whereas the barriers are reverse-scored. The higher the respondents’ total score, the higher their perception of exercise. This questionnaire has a validated Shona translation, which was used and has demonstrated good validity and reliability α = 0.8 in a study conducted on people living with HIV [31].

**The EQ-5D-5L** is a generic HRQoL measure consisting of five (5) domains, i.e. mobility, self-care, usual activities, pain/discomfort, and anxiety/depression [32]. The respondent must choose one of the five responses: no problems, slight problems, moderate problems, severe problems, and extreme problems; the responses are weighted one to five, respectively. The instrument also has a visual analogue scale (VAS) score where participants must score their current health status from 0–100, where 0 is the worst health and 100 is the best health. It is a validated tool translated to Shona, the local language, with excellent validity and reliability [32].

**The demographic questionnaire** was designed to capture potential covariates. This questionnaire elicited participant demographic characteristics (e.g., education, employment status, and financial adequacy), obstetrics (e.g., parity, trimester timing), and exercise characteristics (e.g., perceived pre-pregnancy and current PA levels).

### Data analysis

Descriptive statistics such as frequencies, percentages, quartiles, median and means were computed to describe participant characteristics and standardized outcomes. Logistic regression was used to evaluate factors associated with physical activity. We used the WHO criteria because there is no normative data for the PPAQ (primary outcome measure). Physical inactivity was defined as spending less than 150 minutes of moderate-intensity physical activity per week [4]. Crude odd ratios were calculated, and all variables yielding values p ≤0.20 were fed into the multivariate binary logistic model. Tests were conducted using SPSS Version 23 at α = 0.05.

## Results

### Sociodemographic characteristics

Most participants had attained secondary level (86.5%), married (92.5%), unemployed (46.6%), perceived their finances as inadequate (53.6%), considered their food security as optimal (40.4%), and reported optimal to good health status (75.8%). Further, most pregnant women received no support in household chores (58.2%), with family members or relatives (46.8%) providing support where support with chores was available. Overall, participants rated the extent of social support as neutral/optimal (41.0%). Last, most participants preferred group (39.7%) or individual exercise regimens (37.5%), exercise through dancing (32.7%) and exercising at home (66.9%)–**See Table 1**.

**Table 1:** Sociodemographic characteristics, N = 517.

Variable	Attribute	Frequency, n (%)
*Age	Mean (SD) years	26.1 (5.9)
Education	Primary	31 (6.0)
	Secondary	447 (86.5)
	Tertiary	39 (7.5)
Marital Status	Married	478 (92.5)
	Single	24 (4.6)
	Other	15 (2.9)
Employment	Unemployed	241 (46.6)
	Housewife	184 (35.6)
	Student	15 (2.9)
	Informal	43 (8.3)
	Formal	34 (6.6)
Financial adequacy	Very inadequate	110 (21.3)
	Inadequate	167 (32.3)
	Somewhat adequate	206 (39.8)
	Adequate	29 (5.6)
	Very adequate	5 (1.0)
Food security	Very inadequate	54 (10.4)
	Inadequate	127 (24.6)
	Neutral	209 (40.4)
	Adequate	106 (20.5)
	Very adequate	21 (4.1)
Health Status	Extremely poor	25 (4.8)
	Poor	43 (8.3)
	Optimal	180 (34.8)
	Good	212 (41.0)
	Very Good	57 (11.0)
Household assistance	Yes	216 (41.8)
	No	301 (58.2)
Source of social support	Maid/helper	35 (16.2)
	Spouse/partner	85 (39.4)
	Relative/family	101 (46.8)
Exercise preference	Individual	194 (37.5)
	Group	205 (39.7)
	Combined	118 (22.8)
Preferred exercise method	Walking slowly	141 (27.3)
	Dancing	169 (32.7)
	Prenatal exercise class	106 (20.5)
	Walking quickly	111 (21.5)
	Running slowly	22 (4.3)
	Running fast	15 (2.9)
	Other	10 (1.9)
Preferred exercise place	Gym	67 (13.0)
	Home	346 (66.9)
	Clinic	45 (8.7)
	Community	31 (6.0)
	Local school	17 (3.3)
	Other	9 (1.7)

* Data not in the n (%) format

### Obstetric and health characteristics

Most participants: had been pregnant before (63.4%), were multiparous (71.0%), knew the duration of their pregnancy (93.6%), did not experience complications with the current (80.7%) or previous pregnancy (80.2%), had no chronic condition (87.6%), pregnancy was planned (65.6%) and took less than an hour to travel to the clinic (63.1%)—**Table 2**.

**Table 2:** Obstetric and health characteristics, N = 517.

Variable	Attribute	Frequency, n (%)
First Pregnancy	Yes	189 (36.6)
	No	328 (63.4)
Pregnancy frequency	Primiparous	93 (29.0)
	Multiparous	228 (71.0)
Pregnancy duration awareness	Yes	484 (93.6)
	No	33 (6.4)
Complications current pregnancy	Yes	100 (19.3)
	No	417 (80.7)
Complications previous pregnancy	Yes	65 (19.8)
	No	263 (80.2)
Chronic condition	Yes	64 (12.4)
	No	453 (87.6)
Planned pregnancy	Yes	339 (65.6)
	No	178 (34.4)
Social support	Inadequate	156 (30.2)
	Neutral	212 (41.0)
	Adequate	149 (28.8)
Travel Distance to the clinic	< 1 hour	326 (63.1)
	≥ 1 hour	191 (36.9)

### Exercise habits of pregnant women

Most participants; exercised before pregnancy (58.6%), had decreased PA levels during pregnancy (55.3%), and often exercised with their partner(s) (53.2%). Also, most participants received some exercise advice (64.6%), were advised on individual exercise (51.0%), and received exercise information from social media (36.6%) and nurses (35.8%)—**See Table 3**.

**Table 3:** Exercise Habits, N = 517.

Variable	Attribute	Frequency, n (%)
Exercise pre-pregnancy	Yes	303 (58.6)
	No	214 (41.4)
Current PA level	Decreased	286 (55.3)
	Remained the same	104 (20.1)
	Increased	127 (24.6)
Exercise with partner	No	175 (33.8)
	Sometimes	275 (53.2)
	Yes	62 (12.0)
	Not applicable	5 (1.0)
Received exercise advice	Yes	334 (64.6)
	No	183 (35.4)
Exercise advice method	Individual	170 (32.9)
	Group exercise	104 (20.1)
	Brochure	65 (12.6)
	Other	10 (1.9)
Exercise information sources	Social media	189 (36.6)
	Radio	83 (16.1)
	Television	96 (18.6)
	Friends/Relatives	136 (26.3)
	Newspaper	18 (3.5)
	Public health facility	117 (23.0)
	Private health facility	22 (4.3)
	Other	31 (6.0)
Exercise advice source	Nurse/midwife	185 (35.8)
	Doctor	73 (14.1)
	Physiotherapist	47 (9.1)
	Lay health worker	63 (12.2)
	Other	46 (8.9)

### Physical activity summative indices

The prevalence of physical inactivity in the study participants was 89.9%, based on WHO recommendations of 150 minutes of PA per week. Most study participants engaged in moderate-intensity PA (41.9%), with a few engaging in vigorous-intensity PA (1.9%). Last, pregnant women primarily engaged in household activities (51.1%) and were hardly involved in sports (7.8%)**—See Table 4**.

**Table 4:** Physical activity summative indices, N = 517.

Attribute	Mean (SD)	Median	Range [minimum—maximum]	IQR [lower quartile–upper quartile]
**Intensity (MET-h-week)**				
Sedentary	58.8 (45.4)	49.9	225 [0.0–225]	9.0 [21.0–30.0]
Light	100.2 (62.3)	92.2	363 [0.0–363]	66.4 [21.1–87.5]
Moderate	118.7 (120.9)	79.4	788 [0.0–788]	82.3 [52.2–134.5]
Vigorous	5.5 (8.2)	1.8	41 [0.0–41]	116.2 [36.4–152.6]
Total	283.2 (196.1)	241.6	1410 [5.6–1416]	215.7 [148.3–364.1]
**Activity (MET-h-week)**				
Household	123.1 (86.0)	104.5	565 [0.0–565]	113.5 [59.0–172.5]
Occupational	66.5 (91.5)	30.1	577 [0.0–577]	90.9 [0,0–90.9]
Sports	18.7 (20.6)	12.1	133 [0.0–133]	20.1 [5.1–25.2]
Transport	30.7 (31.0)	20.1	168 [0.0–168]	30.6 [11.4–42.0]

### Exercise benefits and barriers

The most highly perceived benefits of exercise were improved muscle strength (72.1%), mental alertness (70.4%), overall improved mental health (74.3%) and a source of good entertainment (70.6%). The most highly perceived barriers were cost (61.1%) and time pressure, i.e., time taken from family responsibilities (65.8%)–**See Table 5**.

**Table 5:** EBBS frequencies, N = 517.

Classification	Question	Strongly disagree, n (%)	Disagree, n (%)	Strongly agree, n (%)	Agree, n (%)
**Benefits**					
Physical performance	Increases muscle strength	3 (0.6)	34 (6.6)	366 (70.8)	114 (22.1)
	Increases physical fitness	3 (0.6)	27 (5.2)	343 (66.3)	144 (27.9)
	Improves muscle tone	5 (1.0)	40 (7.7)	373 (72.1)	99 (19.1)
	Improves cardiovascular function	4 (0.8)	47 (9.1)	350 (67.7)	116 (22.4)
	Improves body functioning	6 (1.2)	43 (8.3)	362 (70.0)	106 (20.5)
	Improves the way my body looks	6 (1.2)	44 (8.5)	361 (69.8)	106 (20.5)
	Increases stamina	6 (1.2)	36 (7.0)	367 (71.0)	108 (20.9)
Life enhancement	Sleep better	4 (0.8)	53 (10.3)	348 (67.3)	112 (21.7)
	Decreases fatigue	6 (1.2)	54 (10.4)	343 (66.3)	114 (22.1)
	Mental alertness	4 (0.8)	38 (7.4)	364 (70.4)	111 (21.5)
	Carry out normal activities	7 (1.4)	53 (10.3)	354 (68.5)	103 (19.9)
Psychological outlook	I enjoy exercise	13 (2.5)	46 (8.9)	356 (68.9)	102 (19.7)
	Decreases stress and tension	4 (0.8)	44 (8.5)	365 (70.6)	104 (20.1)
	Improves mental health	4 (0.8)	36 (7.0)	384 (74.3)	93 (18.0)
	Makes me feel relaxed	8 (1.5)	101 (19.5)	330 (63.8)	78 (15.1)
Social interaction	Contact with friends	15 (2.9)	126 (24.4)	316 (61.1)	60 (11.6)
	Meet new people	11 (2.1)	104 (20.1)	347 (67.1)	55 (10.6)
	Good Entertainment	11 (2.1)	67 (13.0)	365 (70.6)	74 (14.3)
	Increases acceptance	15 (2.9)	114 (22.1)	332 (64.2)	56 (10.8)
**Barriers**					
Time expenditure	Takes too much of my time	28 (5.4)	120 (23.2)	339 (65.6)	30 (5.8)
	Takes too much time from family	25 (4.8)	119 (23.0)	339 (65.6)	34 (6.6)
	Takes too much time from family responsibilities	20 (3.9)	124 (24.0)	340 (65.8)	33 (6.4)
Exercise environment	Facilities have inconvenient schedules	36 (7.0)	205 (39.7)	248 (48.0)	28 (5.4)
	Costs too much	25 (4.8)	103 (19.9)	316 (61.1)	73 (14.1)
	Exercise clothes look funny	37 (7.2)	150 (29.0)	293 (56.7)	37 (7.2)
	Too few places to exercise	38 (7.4)	200 (38.7)	233 (45.1)	46 (8.9)

### Exercise benefits barriers summative indices

The highest perceived benefit was life enhancement, with a mean score of 12.4 (SD = 1.8). The barriers subscale had a mean of 18.8 (SD = 3.4), with the highest perceived barrier being the exercise environment, with a mean of 10.6 (SD = 2.1)—**see Table 6**.

**Table 6:** EBBS summative indices, N = 517.

Variable	Mean (SD)	Median	Range [minimum-maximum]	IQR [Lower quartile-upper quartile]
**Benefits**				
*Life enhancement*	12.4 (1.8)	12.0	9 [7.0–16.0]	1.0 [12.0–13.0]
*Psychological outlook*	12.2 (1.7)	12.0	12 [4.0–16.0]	1.5 [11.5–13.0]
*Social interaction*	11.5 (2.0)	12.0	12 [4.0–16.0]	2.0 [10.0–12.0]
*Benefits Subscale*	57.9 (7.0)	57.0	46 [30.0–76.0]	6.0 [55.0–61.0]
**Barriers**				
*Time expenditure*	8.2 (1.7)	9.0	9 [3.0–12.0]	2.0 [7.0–9.0]
*Exercise environment*	10.6 (2.1)	11.0	12 [4.0–16.0]	3.0 [9.0–12.0]
*Barriers Subscale*	18.8 (3.4)	19.0	21 [7.0–28.0]	4.0 [17.0–21.0]

### Health-related quality of life (HRQoL)

Most participants had no problems with mobility (61.3%), self-care (79.3%), usual activities (72.3%), or anxiety or depression (67.5%), with a few participants reporting slight pain and discomfort (32.9%)–**See S1 Table**. The mean EQ-5D utility and VAS scores were .80 (SD 0.1) and 78.2 (SD 22.9), respectively; these indices reflect high HRQoL evaluations–**See S2 Table**.

### Binary logistic regression

In the bivariate analysis, these variables were associated with physical inactivity: lower education, employment status (being unemployed), first pregnancy, not habitually exercising pre-pregnancy, perceived current PA activity levels, source of exercise advice, exercise information source, preferring individual exercise, preferred exercise method, preferred exercise location, financial inadequacy, and travelling less than an hour to the clinic–**See S3 Table**. After adjustment for confounding and covariance using binary logistic regression, these variables remained statistically significant and were associated with physical inactivity; lower education i.e. primary (AOR 5.24 [1.69: 16.19]), multigravida (AOR .47 [.31: .42]), not exercising before pregnancy PA (AOR 2.02 [1.30: 3.13]), perceived decreased current PA level (AOR 2.04 [1.22: 3.43]) and not being advised by a doctor on exercise (AOR 2.05 [1.04: 4.04])–**See Table 7**.

**Table 7:** Adjusted odds ratios.

Variable	Attribute	AOR [95% CI]	p-value
*Education*	Primary	5.24 [1.69: 16.19]	.004*
	Secondary	2.10 [.97: 4.54]	.059
	Tertiary (ref)		
*First pregnancy*	No	.47 [.31: .42]	.001*
	Yes (ref)		
Exercise pre-pregnancy	No	2.02 [1.30: 3.13]	.002*
	Yes (ref)		
*Current PA level*	Decreased	2.04 [1.22: 3.43]	.007*
	Remained the same	1.42 [.79: 2.57]	.239
	Increased (ref)		
*Exercise with partner*	No	8.87 [.73: 108.57]	.088
	Sometimes	7.61 [.62: 93.24]	.112
	Yes	3.24 [.25: 41.87]	.368
	Not applicable (ref)		
*Exercise advice*	No	2.53 [.08: 80.99]	.599
	Yes (ref)		
*Exercise advice method: individual*	No	0.40 [.15: 1.09]	.074
	Yes (ref)		
*Exercise advice method: group*	No	0.39 [.14: 1.04]	.059
	Yes (ref)		
*Exercise advice method: just information*	No	0.80 [.27: 2.40]	.691
	Yes (ref)		
*Exercise information source: social media*	No	1.01 [.65: 1.58]	.954
	Yes (ref)		
*Exercise information source: radio*	No	.96 [.56: 1.64]	.873
	Yes (ref)		
*Exercise information source: TV*	No	1.70 [1.00: 2.90]	.052
	Yes (ref)		
*Exercise information source: friends, relatives*	No	.66 [.41: 1.06]	.083
	Yes (ref)		
*Exercise information source: newspaper*	No	1.16 [.39: 3.51]	.789
	Yes (ref)		
*Exercise information source: public health facility*	No	1.43 [.85: 2.40]	.171
	Yes (ref)		
*Exercise information source: private health facility*	No	2.25 [.79: 6.37]	.128
	Yes (ref)		
*Exercise advice source: Nurse*	No	.66 [.38: 1.15]	.145
	Yes (ref)		
*Exercise advice source: Doctor*	No	2.05 [1.04: 4.04]	.037*
	Yes (ref)		
*Exercise advice source: Physiotherapist*	No	1.10 [.50: 2.43]	.815
	Yes (ref)		
*Exercise advice source: Lay health worker*	No	.63 [.32: 1.25]	.186
	Yes (ref)		
*Social support*	Inadequate	1.06 [.63: 1.79]	.882
	Neutral	1.16 [.72: 1.86]	.544
	Adequate(ref)		
*Benefits subscale*		.96 [.93: .99]	.004*

* Denotes statistically significant result

## Discussion

This study sought to determine the level of physical activity and its associated factors in pregnant women attending primary healthcare facilities in Harare, Zimbabwe. Most of the pregnant women were physically inactive and engaged in primarily moderate-intensity PA and household activity. Lower education, not exercising before pregnancy, multigravida, and not being advised by a doctor on exercise increased the odds of being physically inactive.

### Physical inactivity prevalence

This study showed a high prevalence of physical inactivity, with 89% of pregnant women not meeting the WHO recommendations of engaging in at least 150 minutes of moderate-intensity physical activity per week [4]. This high prevalence of sedentary behaviors in pregnancy is comparable to cross-sectional studies conducted in pregnant women in Ethiopia (N = 299) and China (N = 1056), yielding physical inactivity prevalence of 79.3% and 88.9%, respectively, using the PPAQ [12, 15]. In the Ethiopian study, the high prevalence of sedentary behavior was accredited to modernization and urbanization; this applies to the current study setting. The SSA region has seen an upsurge of non-communicable diseases primarily driven by lifestyle changes, including sedentary lifestyles/behaviors (e.g. increased motorized locomotion), dietary changes (e.g. consumption of processed foods) and lifestyle changes (e.g. increased substances and alcohol intake) [12, 33]. For the Chinese study, physical inactivity was hugely driven by safety concerns (e.g., fear of miscarriage) and cultural myths. In the Chinese culture, pregnancy is considered a vulnerable time and pregnant women are discouraged from engaging in PA as it is considered taboo and the belief that not exercising is associated with increased fetus growth [15]; these beliefs are also prevalent in Zimbabwe. Elsewhere, a South African cross-sectional study (N = 1082) using the PPAQ also yielded a high physical inactivity prevalence, as 74.3% of the pregnant women were physically inactive, primarily driven by a lack of resources [29]. Previous systematic reviews have shown a link between poverty and physical inactivity [34]. For example, a lack of access to safe exercising spaces and human resources (e.g. public/government-sponsored fitness instructors) is a massive barrier to engagement in structured physical activity in pregnant women in SSA [29, 35]. For instance, in the current study, a lack of public exercise facilities was primarily seen as a barrier to engagement in regular and safe physical activity during pregnancy. In this study, most participants were unemployed, a trend like most SSA countries and were thus likely to afford private exercise facilities. In contrast, in high-income countries with robust public exercise facilities, other barriers such as fatigue, lack of time, motivation and confidence, not cost, have been reported to impact pregnant women’s ability to engage in optimal PA [35].

Contrastingly, a Malaysian cross-sectional study (N = 339) conducted on pregnant women in their first trimester yielded a 38.3% physical inactivity prevalence using PPAQ [2], significantly lower than our study. The Malaysian study consisted only of pregnant women in their first trimester, which may explain the lower prevalence of physical inactivity [2]. Pregnant women are more likely to exercise more in the first trimester due to fewer physical complaints (e.g. pain and discomfort) and reduced fears of miscarriage when compared to exercising more in the second and third trimesters [36, 37]. Elsewhere, an Ethiopian cross-sectional study (N = 442) using PPAQ reported an even lower prevalence of physical inactivity of 21.9% [11]. The Ethiopian study primarily recruited pregnant women from rural areas, unlike our study, which focused on pregnant women from urban areas. Pregnant women in rural areas are more likely to be exposed to more laborious chores (e.g., fetching water and firewood). They are likely to walk more distance, unlike urban counterparts who are most likely to use motorized transportation, hence discrepancies in PA levels [11]. Despite the variable PA prevalence estimates, it seems reasonable to conclude that pregnant women in low-resourced settings are at high risk of physical inactivity. Multi-sectorial efforts are needed to optimize PA during pregnancy for the realization of the health benefits associated with regular PA engagement.

### Physical activity subdomains

The prevalence of moderate-intensity PA (41.9%) was much higher than studies from comparable settings [12, 29, 38]. For instance, in Ethiopian (N = 299), Brazilian (N = 1279) and South African (N = 1082) cross-sectional studies, only 20.6%, 23.1% and 18.7% of participants engaged in moderate-intensity PA, respectively, per PPAQ criteria [12, 29, 38]. In our setting, most women were likely to perform several manual household chores (e.g., manual laundry), hence a higher prevalence of moderate-intensity PA. Similarly, a Malaysian study revealed a high prevalence of household chores, which accounted for most moderate-intensity energy expenditure [2]. More importantly, most participants in our study preferred walking (48.8%) as a form of exercise; this is consistent with resource availability and partially accounts for PA behaviors in Zimbabwean pregnant women. Most women in our study were unemployed and reported inadequate finances. They were more likely to walk than use motorized transportation, particularly over short to medium distances, due to the high cost of motorized transportation owing to the ongoing economic challenges. Our findings are consistent with a South African study, which reported 76.9% and 80.0% prevalence rates of household chores and walking, respectively [5].

In this study, most participants spent the most time on household activity, with a prevalence of 62.6%. In a study on South African pregnant women (N = 1082) where PPAQ was used, the prevalence of household activity was 48.6% [29]. This is much lower than our study; however, this is unsurprising as almost 36% of pregnant women were homemakers due to ongoing socioeconomic challenges. Like findings from this study, a cross-sectional study done in Nigeria (N = 453) using PPAQ yielded a 57.7% prevalence of household activities. Pregnant women in the Nigerian study felt safe engaging in household PA, and more than half of the participants were reported to be in their third trimester, where maternity leave begins; hence, they were more likely to spend more time at home [28]. However, our results differ from those of a cross-sectional study conducted in China (N = 1077). Using the PPAQ, occupational activity contributed the most to the total PA expenditure, with a prevalence of 53.7% [39]. This difference may be attributed to the higher percentage of employed women in China compared to our study, where most participants were unemployed (46.6%) and housewives (35.6%); hence, their PA was mostly household work. The high prevalence of household activity across studies may be accounted for by the fact that, during pregnancy, women tend not to engage in any other activity besides housework, which they consider safe [40]. In Zimbabwe culture, women, including those who are pregnant, are expected to carry out their maternal duties, which entail mainly housework, hence the high level of household activity [41].

### Factors associated with physical inactivity

In this study, pregnant women with lower levels of education had higher odds of being physically inactive. For example, women with primary education were five (5) times more likely to be physically inactive than those with tertiary education. Congruent to an Ethiopian cross-sectional study (N = 299), women with no or little formal education were 13 times more likely to be physically inactive compared to those with high levels of formal education (AOR: 13.50, 95% CI: 2.65–68.91) [12]. Pregnant women with lower levels of education may have limited knowledge of the harmful effects of sedentary behaviors during the gestation period [12, 34, 42]. The high prevalence of myths regarding the harmful effects of physical activity (e.g. fear of miscarriage) is testimony to the link between low education and health behavior [12, 15]. Conversely, educated mothers are more likely to be exposed to multiple information sources (e.g. the internet) and are likely to be more knowledgeable on the benefits of PA, enabling them to make more health-conscious decisions [43]. Therefore, there is a need for conscious efforts by healthcare professionals to disseminate information on the benefits of PA among pregnant women. Health education and promotion are low-hanging first-level interventions for tackling the vast burden of physical inactivity in pregnancy.

Pregnant women who exercised with their partners were more likely to have increased odds of being physically inactive than those who did (AOR: 8.87, 95% CI: .73–108.57). Similarly, a Chinese study (N = 1056) reported that pregnant women who exercised regularly with their spouses were more likely to be physically active (AOR: 2.21, 95% CI: 1.33–3.67) [15]. Regular social support is essential for the uptake and maintenance of regular physical activity during pregnancy [44, 45]. Social support, in its many forms, is a buffer to stressful life events, in this case, the psychosocial and physical challenges associated with pregnancy [45]. For example, spousal social support is essential for constant motivation to fight off pregnancy-associated fatigue and bodily pain [45]. Elsewhere, a cross-sectional survey of Iranian women (N = 300) found that family discouragement was the highest reported perceived barrier to PA engagement [10]. In Iranian women, social support was a salient source of emotional and informational support for diet and physical activity-related beliefs and behaviors among pregnant women, and this finding is further supported by a systematic review [35]. Even in non-pregnant women, encouragement and companionship are essential for optimal adherence to daily PA routines [45, 46]. Our study also shows the importance of informational support. For instance, receiving exercise advice from a doctor was associated with regular physical activity engagement in pregnancy. Similar to our study, a Brazilian study (N = 3580) demonstrated that pregnant women receiving advice from a health professional had increased PA engagement than those without (AOR: 1.82, 95% C1: 1.37–2.42) [47]. In Zimbabwean society, doctors and healthcare professionals are perceived as high members of the society whose advice is highly esteemed. Collectively, there is a need for interventions that foster social support by actively engaging the families and partners of pregnant women to increase the uptake and maintenance of adequate PA during pregnancy. Importantly, there is a need for health professionals to step up health promotional efforts (e.g., exercise counselling) to increase PA in pregnancy.

Consistent with a previous systematic review [34], this study also revealed that women who were not physically active before pregnancy were twice (AOR: 2.02, 95% CI: 1.30–3.13) as likely to be physically inactive during pregnancy. Similarly, in a study conducted on Brazilian pregnant women (N = 1279), respondents who regularly exercised before pregnancy were six (6) times more likely to be physically active during pregnancy (OR = 6.45; CI 95% 4.64–8.96) [38]. Women who exercise before pregnancy are more likely to be more knowledgeable about the benefits of PA. Importantly, regular PA engagement is a habitual positive health behavior, and pregnant women with high pre-pregnancy PA levels are likely to find a great impetus to continue exercising during pregnancy [38].

Multigravida women were more likely to be physically active (AOR .47 95% CI: .31-.42). Similarly, in Malaysia (N = 339) and Ethiopia (N = 299) being primigravida was a determinant of physical inactivity, which was attributed to first-time mothers being more cautious and apprehensive of engaging in PA [2, 12]. Multigravida women are more likely to be physically active as they have lived experiences of pregnancy and other children to care for, which requires them to be more active [28].

In this study, the exercise environment and cost were the most salient perceived barriers to PA engagement in pregnant women. These findings mirror outcomes of a systematic review that reported lack of facilities and affordability as salient environmental barriers to PA engagement in pregnant women [35]. Due to a lack of access to public exercise facilities, most participants were most unlikely to afford private gym facilities memberships or prenatal classes due to the economic situation in the country. More importantly, the lack of a safe environment, for example, street lighting or safe spaces for regular physical activity, was a barrier to PA engagement. Systematic reviews identified environmental factors (e.g. lack of safe spaces), lack of time, discomfort during pregnancy and pain as the salient barriers to PA engagement in pregnancy [34, 35]. Therefore, there is a need for multi-sectoral approaches to enhance pregnant women’s propensity to exercise. For example, environmental changes such as safe walking lanes and public spaces harnessing green spaces’ therapeutic effects may increase pregnant women’s regular PA engagement.

### Strengths and limitations

The strengths of our study included the recruitment of a large sample size, the study being conducted in Zimbabwe and the use of validated outcome measures. However, the PPAQ is a self-reported tool whose validity and reliability depend on the participant’s ability to recall their physical activity patterns across semesters; the chances of recall and social desirability biases are very high. Future studies should concurrently use objective measures (e.g., pedometers) to verify self-report PA evaluations. Also, causality could not be inferred as we used a cross-sectional study design. Future studies, particularly prospective cohorts recruiting pregnant women from the first semester and following them up through the postnatal period, will provide significant insights into PA engagement behaviors across the pregnancy trajectory. Lastly, participants in this study were recruited from public health care centers only, which may preclude the study from selection bias. Future studies should recruit participants across diverse settings, such as communities and non-health facilities.

## Conclusion

Our study revealed a high prevalence of sedentary behavior among Zimbabwean pregnant women. Multi-sectorial efforts are needed to optimize PA during pregnancy. For instance, health education and promotion efforts using public health approaches and individualized exercise counselling are low-hanging interventions for tackling the colossal burden of physical inactivity during pregnancy. Importantly, there is a need to consider task-shifting approaches to exercise counselling to increase reach given resource limitations. At the policy level, there is a stern need to provide guidelines and resources to implement interventions that increase PA engagement among pregnant women. For example, there is a need to consider implementing supervised and group-based antenatal exercise classes within primary healthcare facilities and communities to increase uptake, feasibility, and sustainability.

## Supporting information

S1 Table EQ-5D-5L frequencies. (DOCX)

S2 Table EQ-5D-5L summative indices. (DOCX)

S3 Table Bivariate unadjusted logistic regression association between physical inactivity and covariates. (DOCX)

S1 Dataset (XLSX)
