# Supplementary material for: Predictors of physical activity among pregnant women in Harare, Zimbabwe
Source: PLOS Glob Public Health. 2025 Jan 6;5(1):e0003470. doi: 10.1371/journal.pgph.0003470 (PMC11703015; doi:10.1371/journal.pgph.0003470)
Supplement: S1 Table — (DOCX) [file pgph.0003470.s001.docx]

# Supporting information 1

## S1 Table : EQ-5D-5L frequencies

Table 1

| **Domain** | **No problems, n (%)** | **Slight problems, n (%)** | **Moderate problems, n (%)** | **Severe problems, n (%)** | **Unable, n (%)** |
| --- | --- | --- | --- | --- | --- |
| Mobility | 317 (61.3) | 100 (19.3) | 73 (14.1) | 21 (4.1) | 6 (1.2) |
| Self-care | 410 (79.3) | 65 (12.6) | 33 (6.4) | 8 (1.5) | 1 (0.2) |
| Usual activities | 374 (72.3) | 102 (19.7) | 29 (5.6) | 10 (1.9) | 2 (0.4) |
| Pain/discomfort | 253 (48.9) | 170 (32.9) | 67 (13.0) | 21 (4.1) | 6 (1.2) |
| Anxiety/depression | 349 (67.5) | 101 (19.5) | 42 (8.1) | 19 (3.7) | 6 (1.2) |
