# Supplementary material for: Predictors of physical activity among pregnant women in Harare, Zimbabwe
Source: PLOS Glob Public Health. 2025 Jan 6;5(1):e0003470. doi: 10.1371/journal.pgph.0003470 (PMC11703015; doi:10.1371/journal.pgph.0003470)
Supplement: S2 Table — (DOCX) [file pgph.0003470.s002.docx]

## S2 Table: EQ-5D-5L summative indices

| **Variable** | **Mean (SD)** | **Median** | **Range [minimum - maximum]** | **IQR [Lower quartile – upper quartile]** |
| --- | --- | --- | --- | --- |
| EQ-5D Utility score | 0.80 (0.1) | 0.8 | 0.7 [0.2 – 0.9] | 0.2 [0.7 – 0.9] |
| EQ-5D VAS | 78.2 (22.9) | 84.0 | 100 [0.0 - 100] | 31.0 [67.0 – 98.0] |
