# Supplementary material for: Predictors of physical activity among pregnant women in Harare, Zimbabwe
Source: PLOS Glob Public Health. 2025 Jan 6;5(1):e0003470. doi: 10.1371/journal.pgph.0003470 (PMC11703015; doi:10.1371/journal.pgph.0003470)
Supplement: S3 Table — (DOCX) [file pgph.0003470.s003.docx]

## S3 Table: Bivariate unadjusted logistic regression association between physical inactivity and covariates

|  | | **Median METs criteria** | | **PA duration criteria/ mins** | |
| --- | --- | --- | --- | --- | --- |
| **Variable** | **Attribute** | **COR [95% CI]** | **p-value < .200** | **COR [95% CI]** | **p-value, < .200** |
| Age |  | 1.03 [1.00: 1.06] | **.095** | 1.02 [.97:1.07] | .489 |
| Education | Primary | 3.17 [1.19: 8.46] | **.021** | .59 [.145:2.43] | .469 |
|  | Secondary | 2.06 [1.03: 4.10] | **.041** | 1.07 [.36: 3.16] | .901 |
|  | Tertiary (ref) |  |  |  |  |
| Marital status | Married | 1.17 [.42: 3.27] | .769 | 1.44 [.32: 6.59] | .637 |
|  | Single | .82 [.22: 2.99] | .759 | .77 [.12: 4.82] | .779 |
|  | Other (ref) |  |  |  |  |
| Employment | Unemployed | 1.51 [.69: 3.33] | .305 | 1.72 [1.02 :2.89] | **.041** |
|  | Housewife | 1.29 [.57: 2.92] | .543 | 1.66 [.96: 2.85] | **.069** |
|  | Formal/informal (ref) |  |  |  |  |
| First pregnancy | No | .73 [.51: 1.04] | **.083** | .55 [.283: 1.05] | **.070** |
|  | Yes (ref) |  |  |  |  |
| Pregnancy duration |  | 1.00 [.99: 1.02] | .619 | 1.00 [.98: 1.01] | .638 |
| Complications current pregnancy | No | 1.32 [.85: 2.05] | **.220** | 1.30 [.655: 2.58] | .453 |
|  | Yes (ref) |  |  |  |  |
| Complications previous pregnancy | No | 1.32 [.76 : 2.29] | .317 | .71 [,28 : 1.76] | .456 |
|  | Yes (ref) |  |  |  |  |
| Chronic condition | No | 1.33 [.59 : 2.96] | .493 | 1.33 [.79 : 2.26] | .286 |
|  | Yes (ref) |  |  |  |  |
| Exercise pre-pregnancy | No | 1.62 [1.14 : 2.30] | **.008** | 1.52 [.83 : 2.79] | **.178** |
|  | Yes (ref) |  |  |  |  |
| Current PA level | Decreased | 1.41 [.93 : 2.15] | **.110** | 2.13 [1.12 : 4.04] | **.021** |
|  | The same | 1.27 [.75 : 2.13] | .371 | 1.97 [.86 : 4.54] | **.110** |
|  | Increased (ref) |  |  |  |  |
| Household assistance | No | 1.03 [.73 : 1.47] | .858 | 1.28 [.71 : 2.32] | .413 |
|  | Yes (ref) |  |  |  |  |
| Source of assistance | Maid | 1.26 [.61 : 2.59] | .540 | 1.43 [.44 : 4.59] | .550 |
|  | Spouse | 1.46 [.85 : 2.53] | **.174** | 1.81 [.70 : 4.65] | .220 |
|  | Relative family | .64 [.37 : 1.09] | .**101** | .38 [.13 : 1.08] | .**070** |
| Exercise with partner | No | 5.28 [.58 : 48.22] | **.140** | 3.38 [.35 : 32.62] | .293 |
|  | Sometimes | 4.15 [.46 : 37.59] | **.206** | 2.21 [.24 : 20.43] | .486 |
|  | Yes | 1.64 [.17 : 15.66] | .669 | 1.16 [.12 : 11.40] | .899 |
|  | Not applicable (ref) |  |  |  |  |
| Exercise Advice | No | 1.38 [.96 : 1.98] | **.081** | 1.15 [.62 : 2.11] | .660 |
|  | Yes (ref) |  |  |  |  |
| Exercise advice method | Individual | .74 [.48 : 1.14] | **.169** | .81 [.40 : 1.63] | .554 |
|  | Group | .80 [.50 : 1.28] | .351 | 1.36 [.66 : 2.81] | .411 |
|  | Brochure | 1.55 [.89 : 2.70] | **.119** | 1.04 [.43 : 2.51] | .925 |
|  | Other (ref) | 3.69 [.77 : 17.65] | **.102** |  |  |
| Source of exercise advice | Nurse/midwife | .64 [.41 : .99] | **.043** | 1.91 [.90 : 4.04] | **.091** |
|  | Doctor | 2.50 [1.44 : 4.36] | **.001** | 1.07 [.47 : 2.47] | .872 |
|  | Physiotherapist | 1.52 [.81 : 2.87] | **.191** | 1.02 [.38 : 2.78] | .963 |
|  | Lay health worker | .77 [.45 : 1.34] | .358 | .70 [.26 : 1.87] | .472 |
|  | Other | .88 [.47 : 1.63] | .678 | .35 [.08 : 1.52] | **.162** |
| Exercise information source | Social media | 1.09 [.76 : 1.56] | .648 | .71 [.34 : 1.48] | .356 |
|  | Radio | .87 [.54 : 1.39] | .549 | .53 [.20 : 1.37] | **.187** |
|  | Television | 1.98 [1.25 : 3.12] | **.004** | 1.20 [.59 : 2.42] | .619 |
|  | Friends/relatives | .60 [.41 : .90] | **.013** | .41 [.18 : .93] | **.033** |
|  | newspaper | .60 [.41 : .90] | **.013** | 2.68 [.85 : 8.46] | **.093** |
|  | Public health facility | 1.33 [.88 : 2.01] | **.175** | 1.26 [.66 : 2.41] | .487 |
|  | Private health facility | 2.21 [.89 : 5.52] | **.089** | 2.07 [.67 : 6.35] | **.206** |
|  | Other | .71 [.34 : 1.48] | .356 | .60 [.14 : 2.59] | .494 |
| Preferred exercise mode | Individual | 1.78 [1.12 : 2.83] | **.014** | 2.21 [1.00 : 4.90] | **.051** |
|  | Group | 1.26 [.80 : 1.99] | .326 | 1.04 [.53 : 2.07] | .905 |
|  | Combined (ref) |  |  |  |  |
| Preferred exercise method | Walking slowly | .69 [.47 : 1.02] | **.061** | .69 [.34 : 1.38] | .295 |
|  | Dancing | 1.17 [.81 : 1.69] | .399 | 1.00 [.54 : 1.84] | .992 |
|  | Prenatal exercise class | 1.37 [.89 : 2.10] | **.156** | 1.51 [.79 : 2.91] | **.217** |
|  | Walking quickly | .96 [.53 : 1.72] | .881 | 1.02 [.38 : 2.69] | .975 |
|  | Running slowly | 1.02 [.67 : 1.56] | .915 | .54 [.24 : 1.23] | **.142** |
|  | Running fast | 1.21 [.51 : 2.85] | .663 | .89 [.20 : 3.91] | .875 |
|  | Other (ref) | 1.52 [.53 : 4.33] | .435 | 6.59 [2.25 : 19.35] | **<.001** |
| Preferred exercise location | Gym | 1.14 [.28 : 4.63] | .852 | 3.43E-9 [4.04E-10 : 2.91E-8] | **<.001** |
|  | Home | 1.35 [.36 : 5.11] | .660 | 5.80E-9 [7.42E-10 : 4.53E-8 ] | **<.001** |
|  | Clinic | 1.20 [.28 : 5.04] | .808 | 2.46E-9 [2.84E-10 : 2.13E-8] | **<.001** |
|  | Community | 1.03 [.23 : 4.58] | .970 | 2.77E-9 [2.96E-10 : 2.59E-8 ] | **<.001** |
|  | Local school | .52 [.10 : 2.79] | .446 | 8.51E-9 [8.51E-9 : 8.51E-9] |  |
|  | Other (ref) |  |  |  |  |
| Financial adequacy | Very inadequate | 4.63 [.50 : 42.74] | **.177** | 13.50 [2.03 : 89.77] | **.007** |
|  | Inadequate | 4.41 [.48 : 40.25] | **.189** | 12.33 [1.93 : 78.82] | **.008** |
|  | Somewhat adequate | 3.49 [.38 : 31.77] | .267 | 16.68 [2.61 : 101.78] | **.003** |
|  | Adequate | 4.29 [.43 :43.14] | .217 | 13.00 [1.51 : 111.78] | **.019** |
|  | Very adequate (ref) |  |  |  |  |
| Food security | Very inadequate | 2.32 [.81 : 6.65] | **.117** | 1.58 [.41 : 6.08] | .506 |
|  | Inadequate | 2.13 [.81 : 5.63] | **.127** | 2.24 [.65 : 7.73] | **.204** |
|  | Somewhat adequate | 2.06 [.80 : 5.31] | **.135** | 2.50 [.76 : 8.22] | **.132** |
|  | Adequate | 1.86 [.69 : 4.96] | **.219** | 2.03 [.58 : 7.13] | .268 |
|  | Very adequate (ref) |  |  |  |  |
| Health status | Extremely poor | .71 [,28 : 1.82] | .473 | .86 [.20 : 3.77] | .844 |
|  | Poor | 1.20 [.54 : 2.68] | .656 | 1.12 [.30 : 4.24] | .870 |
|  | Optimal | 1.01 [.55 : 1.83] | .985 | .94 [.36 : 2.47] | .902 |
|  | Good | .77 [.43 : 1.39] | .391 | 1.20 [.45 : 3.15] | .718 |
|  | Very good (ref) |  |  |  |  |
| Planned pregnancy | No | 1.02 [.71 : 1.46] | .926 | 1.64 [.85 : 3.16[ | **.139** |
|  | Yes (ref) |  |  |  |  |
| Social support | Inadequate | 1.15 [.73 : 1.80] | .557 | 1.21 [.57 : 2.58] | .618 |
|  | Neutral | 1.30 [.85 : 1.98] | **.223** | 1.04 [.53 : 2.05] | .912 |
|  | Adequate(ref) |  |  |  |  |
| Travel distance to clinic | Less than 1 hour | .86 [.60 : 1.23] | .412 | 1.98 [1.11 : 3.52] | **.021** |
|  | An hour or more (ref) |  |  |  |  |
